# Loss of Function of TET2 Cooperates with Constitutively Active KIT in Murine and Human Models of Mastocytosis

**DOI:** 10.1371/journal.pone.0096209

**Published:** 2014-05-02

**Authors:** Serena De Vita, Rebekka K. Schneider, Michael Garcia, Jenna Wood, Mathilde Gavillet, Benjamin L. Ebert, Alexander Gerbaulet, Axel Roers, Ross L. Levine, Ann Mullally, David A. Williams

**Affiliations:** 1 Division of Hematology/Oncology, Boston Children's Hospital, Harvard Medical School, Boston, Massachusetts, United States of America; 2 Division of Hematology, Department of Medicine, Brigham and Women's Hospital, Harvard Medical School, Boston, Massachusetts, United States of America; 3 Institute for Immunology, Medical Faculty Carl Gustav Carus, Dresden University of Technology, Dresden, Germany; 4 Human Oncology and Pathogenesis Program, and Leukemia Service, Department of Medicine, Memorial Sloan-Kettering Cancer Center, New York, New York, United States of America; German Red Cross Blood Service Frankfurt, Germany

## Abstract

Systemic Mastocytosis (SM) is a clonal disease characterized by abnormal accumulation of mast cells in multiple organs. Clinical presentations of the disease vary widely from indolent to aggressive forms, and to the exceedingly rare mast cell leukemia. Current treatment of aggressive SM and mast cell leukemia is unsatisfactory. An imatinib-resistant activating mutation of the receptor tyrosine kinase KIT (*KIT D816V*) is most frequently present in transformed mast cells and is associated with all clinical forms of the disease. Thus the etiology of the variable clinical aggressiveness of abnormal mast cells in SM is unclear. *TET2* appears to be mutated in primary human samples in aggressive types of SM, suggesting a possible role in disease modification. In this report, we demonstrate the cooperation between *KIT D816V* and loss of function of TET2 in mast cell transformation and demonstrate a more aggressive phenotype in a murine model of SM when both mutations are present in progenitor cells. We exploit these findings to validate a combination treatment strategy targeting the epigenetic deregulation caused by loss of TET2 and the constitutively active KIT receptor for the treatment of patients with aggressive SM.

## Introduction

Systemic mastocytosis (SM) is a clonal disease of the mast cell lineage with clinical presentations ranging from mild forms to more aggressive disease [1]. The current classification of SM includes 5 subtypes: indolent SM (ISM), smoldering SM (SSM), SM with an associated hematologic non-mast cell-lineage disease (SM-AHNMD), aggressive SM (ASM), and mast cell leukemia (MCL) [2], [3]. Most cases of SM are mild and respond to symptomatic therapy with antihistamines and inhibitors of mast cell degranulation. Management of aggressive types of disease, where mast cells infiltrate multiple organs (including skin, lymph nodes, spleen, liver, lungs, heart and bone marrow) is more challenging and is approached with chemotherapy or other targeted therapeutic interventions.

In adults, most cases of SM are associated with the presence of activating mutations in the receptor tyrosine kinase *c-KIT* (KIT), which binds to stem cell factor (SCF or KIT ligand), a known trophic factor for mast cells.

By far the most frequent *KIT* mutation in mastocytosis is a substitution of aspartic acid to valine at position 816 *(KIT D816V*) that leads to constitutive activation of the receptor [4], [5]. The presence of the *KIT D816V* mutation does not appear to correlate with a specific subtype of the disease and does not contribute to the disease prognosis. Moreover, this mutation is imatinib-resistant [6], [7] and targeted therapy with second-generation tyrosine kinase inhibitors (TKIs) has shown variable success [8], [9], although promising preliminary data have been obtained with midostaurin (PKC412) [10].

Additional cooperating events may contribute to the pathogenesis and/or the phenotype of SM [11], [12], [13]. Mutations in *TET2* have been reported in as many as 40% of *KIT D816V*-positive SM cases [11], [12]. TET2 is an enzyme that catalyzes the conversion of 5-methylcytosine (5-mC) to 5-hydroxymethylcytosine (5-hmC) and further modified cytosines, regulating gene expression at the cellular level [14], [15]. Loss-of-function mutations in *TET2* have been reported in a variety of hematological malignancies including acute myeloid leukemias (AMLs), chronic myelomonocytic leukemia (CMML), myeloproliferative neoplasms (MPNs), myelodysplastic syndromes (MDS) and lymphoid malignancies [16], [17], [18]. In mouse models, loss of one or both copies of *Tet2* has been shown to contribute to the pathogenesis of hematological malignancies by increasing the self-renewal capacity of the hematopoietic stem cell compartment and expanding the immature pool of myeloid and lymphoid progenitors [19], [20], [21].

In SM, the coexistence of mutations in *TET2* and the *KIT D816V* lesion have recently been suggested to lead to a more aggressive type of disease and an overall worse prognosis, although the effect on mast cell biology was not properly analyzed [22]. In the current study, we investigate the biological relevance of loss of TET2 in the context of *KIT D816V* associated mast-cell disease both *in vivo* in a mouse model and *in vitro* in human cells. We demonstrate that mutations in both loci cooperate in the mast cell lineage to cause an aggressive type of mastocytosis. Furthermore, we show that Tet2 loss-of-function makes *KIT D816V*-positive mastocytosis amenable to combination therapy with epigenetic modifiers and TKIs.

## Methods

### Ethics statement

This study was carried out in strict accordance with the recommendations in the Guide for the Care and Use of Laboratory Animals of the National Institutes of Health. The Institutional Animal Care and Use Commitee at Boston Children's Hospital approved all of the animal care procedures and experiments (authorization no. 11-03-1894R).

### Mice

Kit D814V^Fl^ mice have been previously described [23]. The *Kit D814V* transgene is expressed upon Cre-mediated excision of the loxP-flanked transcriptional stop element in adult mice. Tet2^Fl/Fl^ mice have been described elsewhere [19]. These mice were crossed to Kit D814^Fl^ mice to generate Tet2^Fl/WT^;Kit D814V double transgenic animals, and to Mx1-Cre [24] or Mcpt5-Cre [25] mice to generate Tet2^Fl/WT^;Mx1-Cre and Tet2^Fl/WT^;Mcpt5-Cre double transgenic animals. Tet2^Fl/WT^;Kit D814V were then crossed to Tet2^Fl/WT^;Mx1-Cre or Tet2^Fl/WT^;Mcpt5-Cre mice and their progeny were used for subsequent experiments. In mice carrying the Mx1-Cre allele, Cre expression was induced by 3 i.p. injections of 250 µg polyinosine-polycytidylic acid (pI:C) every second day to activate expression of the transgenic *Kit D814V* and deletion of one or both *Tet2* alleles. Mice were treated with pI:C at 4 weeks of age. All mice used in this study were backcrossed for at least 5 generation and maintained on a C57/BL6 background. All mice were housed in the experimental animal facility at Boston Children's Hospital and were provided free access to food and water.

### Experimental procedures

For survival studies, starting one week after the last pI:C injection, mice were monitored every other day to detect early signs of leukemia. Whenever ruffled fur, reduced movements or hind limb paralysis were noticed, mice were bled by retro-orbital bleeding, and humanely euthanized using carbon dioxide, followed by cervical dislocation. Of the experimental cohorts reported in leukemia studies, all mice were humanely euthanized when they met any of the humane endpoints listed above. One mouse in the primary leukemic mice cohort was lost at follow-up and found dead before humane euthanasia could be performed. WBC counts couldn't be obtained for this animal.

For survival studies and for determination of peripheral blood chimerism, mice underwent retro-orbital bleeding after anesthesia with isofluorane. Following this procedure, animals were treated with eye lubricant to minimize their discomfort.

### Cell lines

The human mast cell leukemia cell line HMC-1.2 (carrying the *KIT G560V* and the *KIT D816V* activating mutations) was a kind gift from Dr. JH Butterfield (Mayo Clinic, Rochester, MN) [26]. HMC-1.2 were grown in RPMI supplemented with 10% fetal bovine serum (FBS), 2 mM glutamine, 100 U/mL penicillin and 0.1 mg/mL streptomycin in a humidified incubator with 5% CO_2_ at 37°C. Fresh HMC-1.2 cells were thawed from an original stock every 8 weeks, and cells were periodically checked for the presence of metachromatic granules and for expression of the KIT receptor [27].

### Derivation of BMMCs

Bone marrow-derived mast cells (BMMCs) were generated from low density BM cells by four weeks of culture in RPMI supplemented with 10% fetal bovine serum (FBS), 2 mM glutamine, 100 U/mL penicillin, 0.1 mg/mL streptomycin and 10 ng/mL of mouse recombinant IL-3 (PeproTech, Rocky Hill, NJ) in a humidified incubator with 5% CO_2_ at 37°C.

### Giemsa staining

Giemsa staining was performed on skin and stomach sections from diseased animals using standard protocols. Slides were evaluated by photomicroscopy using the Spot Advance software from Spot™ Imaging Solutions, (Sterling Heights, MI) on an Eclipse E400 microscope from Nikon (Melville, NY). Images were taken using a 20× objective.

### Cell proliferation and apoptosis

Apoptosis was evaluated using the Annexin V-APC flow kit from BD Biosciences (San Jose, CA). Cells were counterstained with 7-AAD (Invitrogen, Carlsbad, CA). For proliferation assays, cells were cultured in the presence of 5-bromo-2′-deoxy-uridine (BrdU) for 4 hours then fixed, permeabilized, and stained using the APC BrdU Flow Kit (BD Biosciences), following the manufacturer's instructions. Data were acquired on a LSR II flow cytometer (BD Biosciences). For cell growth, cells were plated in 96-well plates at 10,000 cells/well and lysed at the indicated time point with CellTiter-Glo Luminescent Cell Viability Assay reagent (Promega, Madison, WI). Luminescence was read using a DTX 880 plate reader from Beckman Coulter (Brea, CA). Cell growth was normalized to 5 days post-transduction.

### Statistical analysis

Datasets were compared by two-tailed *t* tests and *P* values less than .05 were considered statistically significant.

## Results

### Knock down of TET2 increases proliferation of a *KIT D816V* positive human mast cell leukemia cell line

**Figure 1:**
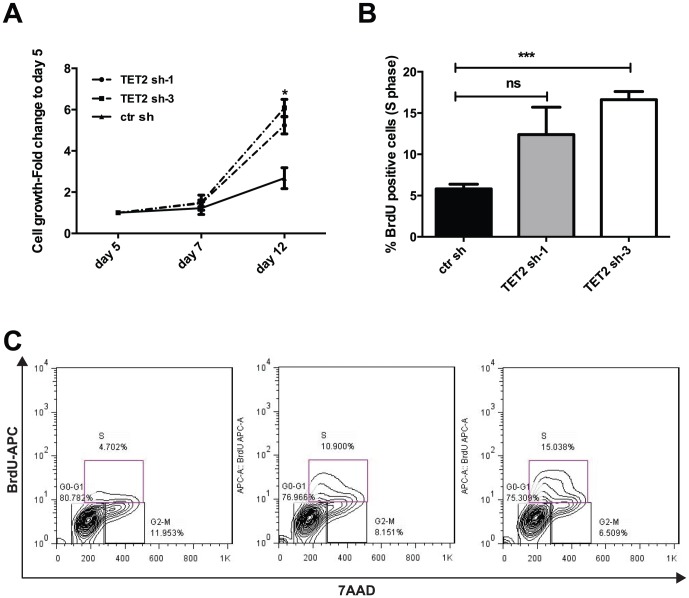
Increased proliferation of HMC-1.2 cells after knock down of TET2. **A**) HMC-1.2 cells were treated with two hairpins against TET2 (TET2 sh-1 and TET2 sh-3) or a control shRNA (ctr sh). Cell growth was calculated using the CellTiter-Glo assay from Promega. Data are presented as fold change relative to day 5 after transduction. Values represent mean ±SEM, n = 3 independent experiments. *P<.05. **B**) Percentage of cells in S-phase determined by BrdU incorporation in HMC-1.2 cells treated with TET2 sh-1 and sh-3 compared to a control hairpin. Values are mean ±SEM. n = 3 independent experiments, ***P<.001, ns =  not significant. **C**) Representative FACS plots showing BrdU incorporation in relation to cell cycle stages in HMC-1.2 cells infected with control hairpin (ctr sh) compared with TET2 sh-1 and TET2 sh-3.

To model the cooperation between loss of function of TET2 and the *KIT D816V* mutation *in vitro*, we knocked down (KD) TET2 in a human mast cell leukemia cell line (HMC-1.2) harboring the *KIT D816V* mutation. We first documented the absence of mutations (except for one annotated SNP) in the coding sequence of *TET2* in the HMC-1.2 cell line (data not shown). In absence of a reliable commercially available antibody for TET2, we determined the efficiency of five individual KD vectors by qPCR (Figure S1, panel A). We chose two vectors (sh-1 and sh-3) that achieved an average KD of TET2 of 45% and 34%, respectively (Table S1). As expected, KD of TET2 in HMC-1.2 cells caused a reduction in the total content of 5-hmC, an intermediate in the DNA demethylation reaction catalyzed by TET2 (Figure S1, panel B). In this cell line, silencing of TET2 also caused a significant increase in cellular growth over time (Fig. 1A) (*P* = .05 TET2 sh-1 vs. control shRNA [ctr sh], *P* = .02 TET2 sh-3 vs. ctr sh, at day 12 after transduction). The increase in cell numbers was associated with increased proliferation upon silencing of TET2, as assessed by BrdU incorporation (Fig. 1B and 1C) (% cells in S phase = 5.8±0.5 ctr sh vs. 12.4±3.3 TET2 sh-1 and 16.63±0.9 TET2 sh-3, *P* = .09 TET2 sh-1 vs. ctr sh, *P* = .0007 TET2 sh-3 vs. ctr). To address whether loss of TET2 would modify migratory properties of HMC-1.2 cells, we compared cells transduced with two shRNAs against TET2 (sh-1 and sh-3) to a ctr sh. There was no difference in the number of migrated cells in response to human SCF under all conditions analyzed (Figure S1, panel C). These data indicate that loss of function of TET2 cooperates with KIT D816V to increase the proliferative capacity of human malignant mast cells, without modifying their migratory properties.

### Tet2^−/−^;Kit D814V mice develop a more aggressive type of systemic mastocytosis compared to Tet2^+/+^;Kit D814V animals

**Figure 2:**
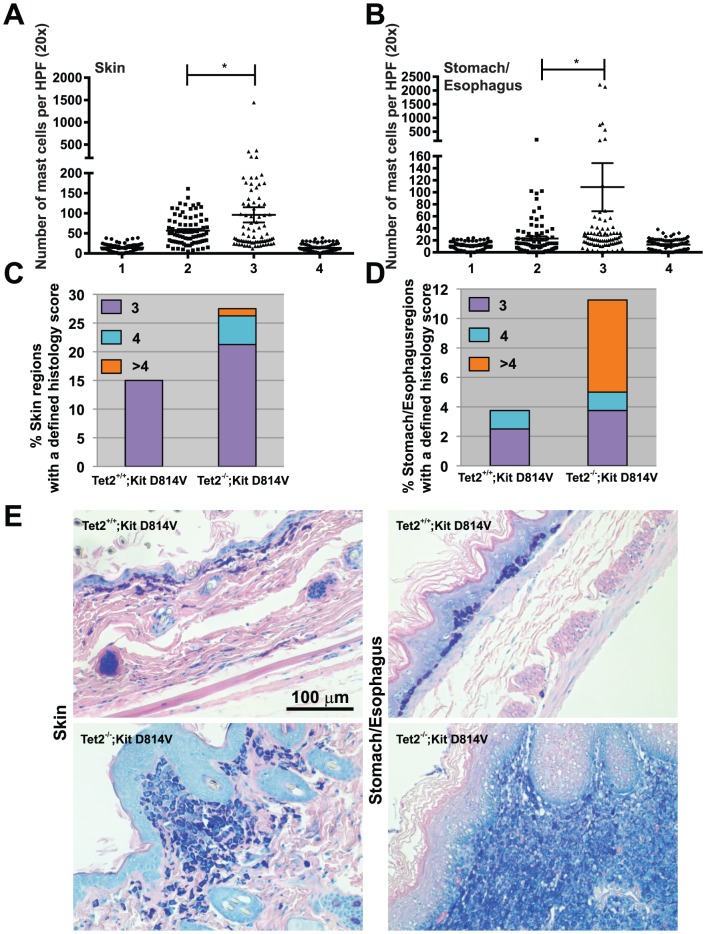
Loss of Tet2 accentuates a Kit D814V driven mast cell phenotype. **A**) Average number of mast cells per skin section across genotypes. N = 60–80 sections from 3–4 independent animals/genotype. *P<.05. **B**) Average number of mast cells per stomach/esophagus section across genotypes. N = 60–80 sections from 3–4 independent animals/genotype. *P<.05. For Figure 2A and 2B, numbers 1–4 indicate the following genotypes: 1 = WT ctr, 2 = Tet2^+/+^;Kit D814, 3 = Tet2^−/−^;Kit D814, 4 = Tet2^−/−^;Kit WT. **C**) Percentage of skin sections with a defined histology score from Tet2^+/+^;Kit D814V and Tet2^−/−^;Kit D814V. **D**) Percentage of stomach/esophagus sections with a defined histology score in Tet2^+/+^;Kit D814V and Tet2^−/−^;Kit D814V animals. For Fig 2A–2D, twenty randomly chosen and independent regions of equal thickness per animal were counted in a blinded fashion at 20× magnification, and scored according to the classification reported in Table 1. Mice were all harvested between 8 and 20 weeks after the last pI:C injection. n = 4 per genotype. **E**) Representative pictures of Giemsa staining performed on skin (left panels) or stomach/esophagus sections (right panels) prepared from Tet2^+/+^;Kit D814V and Tet2^−/−^;Kit D814V animals. Mast cells stain dark blue in these sections. Scale bar represents 100 µm.

Next, we examined the *in vivo* phenotype caused by simultaneous expression of Kit D814V (the mouse homologue of *KIT D816V*) and deletion of Tet2 in the hematopoietic compartment of compound mice. In all genotypes expressing the *Kit D814V* allele there was a significant increase in mast cell infiltration of several organs. In the skin, the average number of mast cells per scored section was 56.9±4 in Tet2^+/+^;Kit D814V vs. 96.3±18.9 in Tet2^−/−^;Kit D814V (n = 80 from 4 independent animals/genotype, *P* = .04, Fig 2A). In the esophagus/stomach, the average number of mast cells per scored section was 23.1±3.6 in Tet2^+/+^;Kit D814V vs. 108.4±40 in Tet2^−/−^;Kit D814V (n = 80 from 4 independent animals/genotype, *P* = .03, Fig 2B). The deletion of Tet2 alone was not sufficient to cause disease in absence of Kit D814V (WT ctr vs Tet2^−/−^; Kit WT *P* = .5 in the skin and *P* = .2, n = 60–80 sections) underscoring the role of Tet2 as a disease modifier, rather than a disease-initiating molecular lesion.

Given that mast cell aggregates ranged in dimension from small infiltrates to mast cell tumors, we defined a histology score (Table 1) to classify and compare disease aggressiveness across areas of affected genotypes. Loss of Tet2 in Kit D814V positive animals was associated with a more aggressive disease phenotype, as evidenced by the presence of histological sections with a score of 4 or greater in target organs (Fig. 2C–2E). These data suggest that simultaneous expression of Kit D814V and loss of Tet2 cooperate in the mast cell compartment and result in a more aggressive phenotype in the skin and the digestive tract than when Kit D814V is present alone.

**Table 1:** Histology score used to classify skin and stomach/esophagus regions from animals with mastocytosis.

Histology Score	Number of mast cells	Description
0	0–30 mast cells	
1	31–50 mast cells	Increased diffuse infiltrate
2	51–100 mast cells	Mast cell accumulation
3	101–200 mast cells	Mast cell tumor<500 µm
4	201–500 mast cells	Mast cell tumor (500–100 µm)
5	501–1000 mast cells	Mast cell tumor (1000–1500 µm)
6	>1000 mast cells	Large tumor>1500 µm

SM is thought to originate from BM stem cells/progenitors that then colonize other sites of disease [28]. The Kit D814V-MxCre model does not develop mast cell infiltration of the BM [23]. However, because Mx-Cre induces deletion of floxed alleles in the BM hematopoietic compartment, we wanted to assess the effect of the Kit mutation alone and of the additional deletion of Tet2 specifically in stem/progenitor cells of the BM, which give rise to mast cells. We therefore performed colony-forming assays and competitive transplants, and found that deletion of Tet2 conferred both increased replating ability to Kit D814V progenitors *in vitro* and a competitive advantage to stem cell/progenitors *in vivo* (Figure S2, panel A and B).

### Loss of one or both copies of Tet2 increases proliferation and impairs differentiation of BMMC carrying the *Kit D184V* mutation

**Figure 3:**
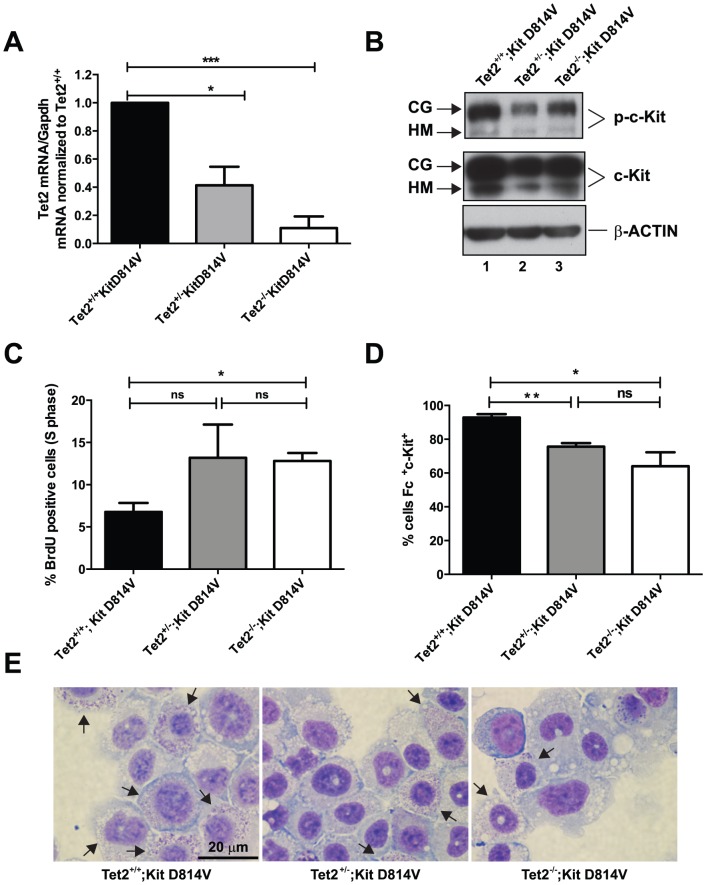
Effects of loss of Tet2 on BMMCs derived from Kit D814V-MxCre mice. **A**) Tet2 mRNA levels measured in BMMCs upon Mx1-Cre-mediated deletion of Tet2. Data are presented as fold change of Tet2 mRNA/Gapdh mRNA levels relative to Tet2^+/+^;Kit D814V. Data represent mean ±SEM, n = 3, *P = <05, ***P<.001. **B**) Phosphorylation of the Kit receptor in the absence of its ligand SCF due to the presence of a Kit activating mutation in BMMCs from Tet2^+/+^;Kit D814V, Tet2^+/−^;Kit D814V and Tet2^−/−^;Kit D814V animals. The western blot shown is representative of two independent experiments with similar results. Two different glycosylated forms of the Kit receptor are indicated. CG = complex glycosylation form; HM = high-mannose form. Total levels of c-KIT and β-actin are shown as loading controls. **C**) Proliferation of BMMCs carrying the *Kit D814V* mutation upon loss of Tet2. Data show average percentage ±SEM of BrdU-positive BMMCs across genotypes, n = 3, *P<.05, ns  =  not significant. **D**) Differentiation of *Kit D814V* positive BMMCs in the absence of Tet2. Data show average percentage ±SEM of double positive (Fcε^+^c-Kit^+^) BMMCs from Tet2^+/+^;Kit D814V, Tet2^+/−^;Kit D814V and Tet2^−/−^;Kit D814V (64.1±8.2) after 4 weeks in culture with mIL-3. n = 3, *P = <05, **P<.01, ns = not significant **E**) Representative images of Tet2^+/+^;Kit D814V,Tet2^+/−^;Kit D814V and Tet2^−/−^;Kit D814V BMMCs. Scale bar  = 20 µm. Arrows indicate cells containing granules, which is indicative of a more differentiated phenotype.

Next, we analyzed the interplay between these two molecular hits during the specification of mast cells from BM progenitors. Given that the identity of mast cell progenitors in adult mice is still controversial [29], [30], we took advantage of an established *in vitro* system that leads to the generation of mature mast cells from bone marrow (BMMCs) upon prolonged culture in the presence of IL-3 [31], [32]. We compared BMMCs from mice harboring the *Kit D814V* mutation, and both, one or no copies of *Tet2* (Tet2^+/+^;Kit D814V, Tet2^+/−^;Kit D814V and Tet2^−/−^;Kit D814V) in the hematopoietic compartment. Excision of the stop cassette allowing expression of the *Kit D814V* transgene and deletion of the *Tet2* floxed allele at the genomic level were verified in BMMCs by PCR (Figure S3, panel A–C). Tet2 mRNA was appropriately reduced according to the genotype (Fig. 3A). Loss of one or both copies of Tet2 did not alter the constitutive phosphorylation of c-Kit caused by the presence of the activating mutation *Kit D814V* in BMMCs (Fig. 3B, lanes 2–4). BMMCs carrying one or no copies of *Tet2* in addition to the *Kit D814V* mutation displayed increased proliferation compared to Tet2^+/+^;Kit D814V cells as measured by BrdU incorporation (Fig. 3C) (% cells in S phase =  6.7% Tet2^+/+^;Kit D814V vs. 13.2 Tet2^+/−^;Kit D814V vs. 12.8 Tet2^−/−^;Kit D814V, n = 3, *P* = .1 for Tet2^+/−^ and .01 for Tet2^−/−^ compared to Tet2^+/+^;Kit D814V,).

Loss of Tet2 also impaired the differentiation of BMMCs. After four weeks in culture with IL-3, 92% of Tet2^+/+^;Kit D814V, but only 75% of Tet2^+/−^;Kit D814V and 64% of Tet2^−/−^;Kit D814V stained double positive for c-Kit and Fcε (co-expression of both markers is indicative of full maturation of mast cells) (Fig. 3D, n = 3, *P* = .001 for Tet2^+/−^ and .02 for Tet2^−/−^ compared to Tet2^+/+^;Kit D814V). The decreased number of double positive cells upon Tet2 deletion was accompanied by a concomitant increase in cells expressing Fcε only (Figure S3, panel D). Morphological analysis of BMMCs revealed that granule formation was reduced upon loss of Tet2 (Fig. 3E, see Tet2^+/+^ in comparison with Tet2^+/−^ and Tet2^−/−^;Kit D814V), suggesting a delayed maturation stage of Kit D814V-Tet2 deleted cells compared to their Tet2 WT counterpart. These morphological changes did not correlate with an altered pattern of expression of a set of mast cell-specific genes (Figure S3, panel E). Together, our data show that loss of function of Tet2 cooperates with Kit D814V to enhance proliferation and alter differentiation of mast cells derived *in vitro* from murine bone marrow progenitors, supporting the hypothesis that the two hits exerts their effect during the specification of mature mast cells from uncommitted progenitors.

### Effect of loss of Tet2 on Kit D814V-driven ALL initiation and progression

**Figure 4:**
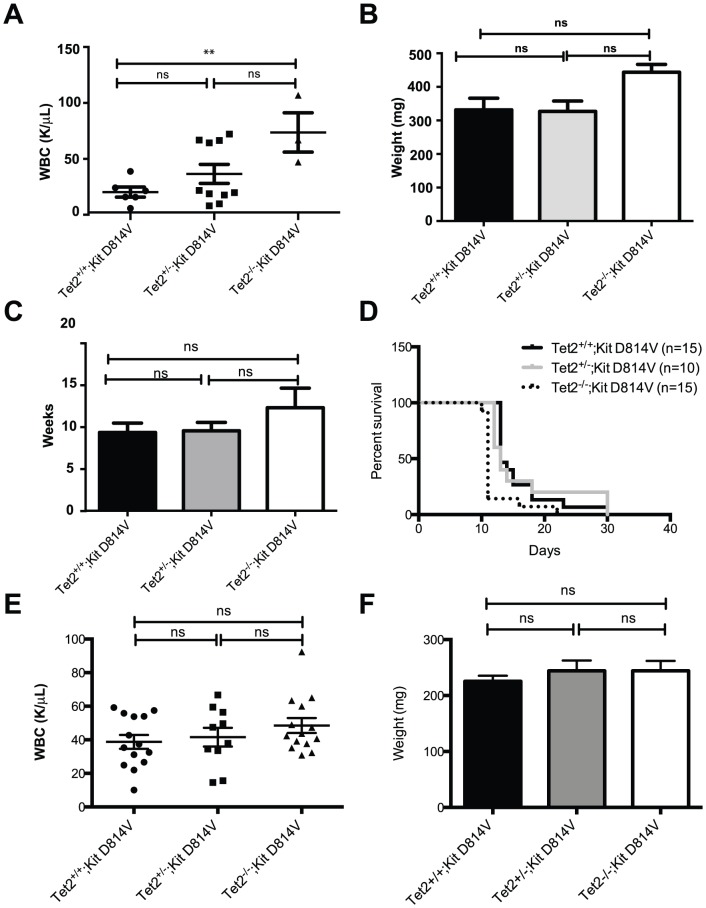
Tet2 deletion affects progression but not initiation of Kit D814V-driven ALL. **A**) Leukocyte counts in moribund leukemic animals according to genotype. Each data point represents an individual animal. **P<.01. **B**) Leukemic infiltration of the spleen expressed as weight (in mg) across genotypes. **C**) Latency of disease (expressed as weeks after the last pI:C injection when mice were found moribund) across genotypes. Data in A B, C is presented as mean ±SEM (n = 3–12 per group). **D**) Kaplan-Meier plot demonstrating survival of recipients transplanted with Tet2^+/+^;Kit D814V (black line),Tet2^+/−^;Kit D814V (grey line) and Tet2^−/−^;Kit D814V (black dotted line) lymphoid leukemic blasts (median survival, 13, 13 and 11 days respectively, n = 10–15 per group, *P* = .009). **E**) Leukocyte counts in secondary moribund leukemic animals according to genotype. Each data point represents an individual animal. **F**) Leukemic infiltration of the spleen expressed as weight (in mg) across genotypes of secondary animals. ns  =  not significant.

As previously reported [23], we noted that Kit D814V;Mx1-Cre transgenic animals induced with pI:C succumb to a disease that resembles human acute lymphoblastic leukemia (ALL). In our experiments, there was no significant difference in the incidence of ALL upon deletion of one or both copies of Tet2 (Table S2). White blood cell counts (WBCs) were significantly higher in Tet2^−/−^;Kit D814V leukemic mice compared with Tet2^+/+^;Kit D814V (Fig. 4A), but there was no significant difference in WBC between Tet2^+/+^;Kit D814V and Tet2^+/−^;Kit D814V mice (Fig. 4B). A trend toward higher spleen weights (a measure of the disease burden in moribund animals) in Tet2^−/−^;Kit D814V mice correlated with higher WBC in these animals, but did not reach statistical difference when compared to Tet2^+/+^;Kit D814V or Tet2^+/−^;Kit D814V animals. There was also no difference across genotypes in disease latency, based on the average time after pI:C when mice were found moribund (Fig. 4C). All mice sacrificed due to ALL had a diffuse generalized increase in cutaneous mast cells (data not shown). Leukemic blasts from all genotypes infiltrated the bone marrow, spleen and liver of diseased animals (Figure S4, panel A). Blast cells in the peripheral blood, marrow and spleen expressed B220 and CD19, suggesting that the leukemia had an immature B cell origin (Figure S4, panel B). Sorted blast cells expressed the *Kit D814V* allele (Figure S4, panel D) and displayed reduction of Tet2 expression consistent with their genotype (Figure S4, panel C), confirming that the leukemic clone harbored both genetic lesions. Upon transplantation into sublethally irradiated recipients, an equal number of blast cells from primary Tet2^+/+^;Kit D814V, Tet2^+/−^;Kit D814V and Tet2^−/−^;Kit D814V mice generated ALL in secondary mice with the same characteristics of the primary disease. There was no difference in the penetrance of disease across the three genotypes in recipient animals, but the median survival was slightly but significantly reduced for recipients of the Tet2^−/−^;Kit D814V group compare with the other two genotypes (median survival for Tet2^+/+^;Kit D814V and Tet2^+/−^;Kit D814V was 13 days, 11 days for Tet2^−/−^;Kit D814V; *P* = .009; Fig. 4D). WBC and spleen weight were comparable across groups of secondary transplanted recipients (Fig. 4E–F). From these data, we conclude that Tet2 is not required for the initiation of Kit D814V-driven acute lymphoblastic leukemia, but may play a role in disease progression in this model.

### Cooperation between the two lesions in a mast cell-specific context

**Figure 5:**
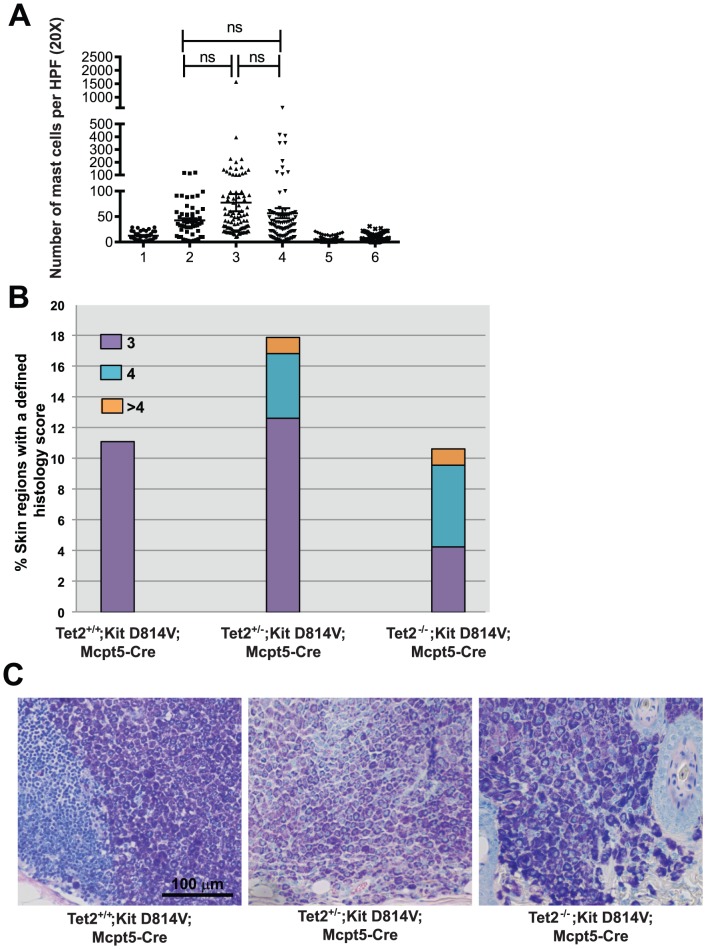
Expression of Kit D814V and loss of Tet2 in MC partially recapitulates the Mx1-Cre-mediated phenotype. **A**) Cutaneous mastocytosis in mice expressing Kit D814V under the control of a mast cell-specific Cre (Mcpt5-Cre) promoter. Graph represents the average number of mast cells/skin section across listed genotypes. N = 56–96 from 3–4 independent animals per genotype. ns = not significant. Numbers 1–6 indicate the following genotypes: 1 = WT ctr, 2 = Tet2^+/+^;Kit D814;Mcpt5-Cre, 3 = Tet2^+/−^;Kit D814;Mcpt5-Cre, 4 = Tet2^−/−^;Kit D814V;Mcpt5-Cre, 5 = Tet2^+/−^;Kit WT;Mcpt5-Cre,6 = Tet2^−/−^;Kit WT;Mcpt5-Cre, **B**) Histological score of disease in affected Mcpt5-Cre animals. Each skin region was scored according to the criteria reported in Table 1. Bar graph represents the percentage of sections per genotype with a defined histology score (from 3 to >4). Mice analyzed were 9-month-old (n = 3–4 per genotype). **C**) Representative microphotographs showing mast cell infiltration in Tet2^+/+^; Kit D814V;Mcpt5-Cre (dermal lymph node), Tet2^+/−^;Kit D814V;Mcpt5-Cre and Tet2^−/−^;Kit D814V;Mcpt5-Cre animals (skin sections). Mast cells stain dark blue in these sections. Scale = 100 µm.

Given the high percentage of mice succumbing to ALL in our Mx-Cre model, we hypothesized that using a mast cell-specific Cre would obviate lymphoid leukemias and allow full penetrance of the mast cell phenotype. Because the Mcpt5 promoter is active selectively in mature mast cells [25], expression of the *Kit D814V* allele driven by this lineage-specific Cre recombinase causes a slow onset (9 months) mastocytosis confined to the skin [23]. In our experiments, the average number of mast cells per skin section was 42.5 in Tet2^+/+^;Kit D814V;Mcpt5-Cre, 77.3 in Tet2^+/−^;Kit D814V;Mcpt5-Cre and 56.5 in Tet2^−/−^;Kit D814V;Mcpt5-Cre (n = 56–96, n = 3–5 animals per genotype). Although there was a trend towards an increased number of mast cells in the skin of Tet2^+/−^;Kit D814V;Mcpt5-Cre and Tet2^−/−^;Kit D814V;Mcpt5-Cre compared to Tet2^+/+^;Kit D814V;Mcpt5-Cre, the difference didn't reach statistical significance (*P* = .1 and .2, respectively). Importantly, the number of mast cells per skin section in Tet2^+/−^;Kit WT;Mcpt5-Cre and Tet2^−/−^;Kit WT;Mcpt5-Cre was not significantly different from the WT control group (Fig 5A), suggesting that in the absence of the Kit D814V lesion, deletion of Tet2 cannot initiate disease in mature mast cells. We also observed that only Tet2^+/−^;Kit D814V;Mcpt5-Cre and Tet2^−/−^;Kit D814V;Mcpt5-Cre animals had aggressive disease as assessed by sections with histology scores >4, according to the classification reported in Table 1 (Fig. 5B and 5C), although the severity of disease varied considerably across skin sections from individual mice. Thus, our data strongly suggest that the cell of origin of the transformed and more aggressive phenotype of mast cell disease likely is a more primitive hematopoietic progenitor and that loss of Tet2 restricted to mature mast cells only modestly accentuates the Kit D814V-driven mast cell skin phenotype.

### Combination therapy with dasatinib and low-dose decitabine in TET2 mutated SM

**Figure 6:**
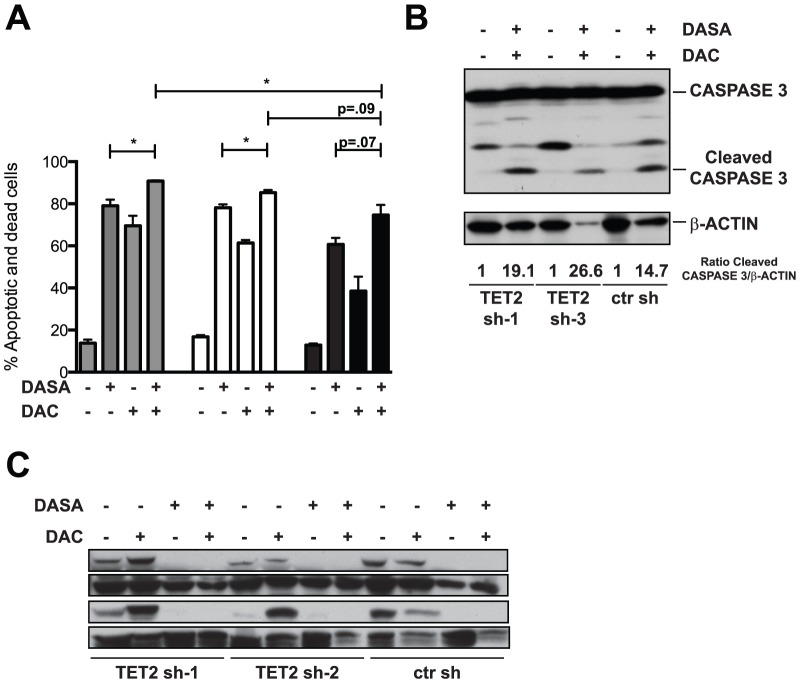
Knock down of TET2 enhances the response of HMC-1.2 to epigenetic modifiers. **A**) HMC-1.2 transduced with two hairpins against TET2 (TET2 sh-1 and TET2 sh-3) or a control shRNA (ctr sh) were treated for 72 hs with low doses of DAC (0.5 µM) or DMSO. After 72 hs, cells were washed and resuspended in media containing DMSO or DASA at a concentration of 1 µM. Annexin V staining was performed 24 hs after the addition of DASA. Bar graph shows percentage of apoptotic cells (Annexin V^+^ 7-AAD^−^) and dead cells (Annexin V^+^ 7-AAD^+^) for each treatment condition. (n = 3; data are expressed as mean ±SEM, *P<.05. **B**) Western blot showing cleavage of CASPASE 3 in HMC-1.2 with or without TET2 shRNA, treated with DAC and DASA or vehicle control. β-actin levels were used as a loading control. Numbers below the β-actin blot indicate the fold change to untreated of the ratio between the cleaved CASPASE 3 band and the loading control, as quantified by densitometry. One of two independent experiments with similar results is shown. **C**) Phosphorylation of SRC and STAT5 after treatment with DASA, with and without pretreatment with DAC. Total SRC and total STAT5 levels were determined to confirm equal loading. One of two or three independent experiments is shown.

Next, we exploited the cooperation between loss of function of TET2 and KIT D816V in the HMC-1.2 cell line to validate possible combinatorial approaches to treatment for ASM and MCL [8], [33]. Loss of TET2 is believed to cause an aberrant methylation of promoter regions in AML [34]. We reasoned that if the same hypermethylator phenotype was caused by loss of TET2 in the *KIT D816V*-positive HMC-1.2 cell line, resulting silencing of gene expression in these cells could potentially be reversed by treatment with epigenetic modifiers, providing an enhanced effect to dasatinib (DASA). We therefore pre-treated HMC-1.2 cells transduced with a control sh or with two independent shRNAs against TET2 with low doses (0.5 µM) of decitabine (DAC) followed by treatment with DASA and performed Annexin V staining. The number of apoptotic (7-AAD^−^/Annexin V^+^) and dead cells (7-AAD^+^/Annexin V^+^) in TET2 KD cells treated with the drug combination was higher than in TET2 KD HMC-1.2 treated with either of the drugs alone (Fig. 6A). In HMC-1.2 cells treated with a ctr sh (TET2 WT), the drug combination induced only a modest effect compared to the TKI alone, due to a lower efficacy of DAC alone in TET2 WT compared to TET2 KD cells (*P* = .02 and *P* = .03 for sh-1 and sh-3 compared to ctr sh treated with DAC alone). Importantly, in the experiments reported here, the number of apoptotic and dead cells was significantly higher in TET2 sh-1 HMC-1.2 cells treated with low doses of DAC followed by DASA than in the control sh group (*P* = .02). Although not reaching statistical significance, there was also a trend towards higher numbers of apoptotic cells in TET2 sh-3 HMC-1.2 cells treated with the drug combination than in the control group (*P* = .09). Treatment with both drugs induced cleavage of CASPASE 3 to a larger extent in TET2 KD sh-1 and sh-3 than in control cells (Fig. 6B, densitometric quantitation of the ratio between cleaved CASPASE 3 and β-Actin expressed as fold change to DMSO treated sample in each condition was: 1 vs. 19.1 in TET2 sh-1, 1 vs. 26.6 in TET2 sh-3 and 1 vs. 14.7 in ctr sh). Furthermore, we observed that, as expected, loss of TET2 did not appear to alter the mechanism of action of DASA, which caused comparable inhibition of phosphorylation of its target SRC kinase and its downstream effector STAT5 in the three conditions (Fig. 6C). We also tested the feasibility of adding DAC to a novel promising TKI, midostaurin (PKC412). Addition of DAC to PKC412 caused only a modest, non-significant increase in the percentage of dead and apoptotic cells compared to the TKI alone (*P* = .07, *P* = .1 and *P* = 3 for TET2 sh-1, sh-3 and ctr sh, respectively), but the number of apoptotic and dead cells was significantly higher in TET2 sh-1 and sh-3 HMC-1.2 cells treated with the two drug-combination than in the control sh group (*P* = .005 and *P* = .01, respectively)(Figure S5). Together, our data suggest that SM with activating mutations in KIT and loss of TET2 is more susceptible to treatment with DAC and DASA than either compound alone, and pretreatment with DAC might also enhance the effect of PKC412 in this setting.

## Discussion

Aggressive forms of systemic mastocytosis (SM) are rare, but difficult to treat. Identifying new molecular targets in aggressive forms of SM may allow for a better stratification of patients and a more effective therapeutic approach. Recent data from primary human samples reported a high frequency of *TET2* mutations in SM and a potential role for loss of function of this gene in modifying the disease phenotype [11], [12], [22]. In this study, we used a combination of *in vitro* and *in vivo* models to investigate a role for TET2 in the pathogenesis of *KIT D816V*-positive systemic mastocytosis.

First, using a model where Kit D814V is expressed in all hematopoietic cells and Tet2 is deleted under the control of an Mx1-Cre promoter, we could conclusively demonstrate that the two mutations cooperate at the level of hematopoietic stem and progenitor cells in the bone marrow. This was evident in functional assays of these cells, including in vitro CFUs assays and in vivo competitive transplants, as wells as during the *in vitro* specification of mast cells from bone marrow progenitors (BMMCs).

In our experiments, loss of Tet2 led to increased proliferation and a block in differentiation of *KIT D814V* positive BMMCs. We found that the coexistence of Tet2 deletion and the *Kit D814V* mutation was insufficient to confer IL-3 independency to BMMCs (data not shown), suggesting that additional genetic alterations are required for transformation of these cells *in vitro*, or that the transforming requirement for these cells *in vitro* might differ from those required to generate a mast cell disease *in vivo*.

The interplay between loss of function of Tet2 and Kit D814V also had specific consequences on the mast cell lineage *in vivo*. We found that loss of Tet2 conferred more aggressive histological features to the SM phenotype in KitD814V transgenic mice, including increased mast cell accumulation in the skin and digestive tract. This observation contributes a possible new mechanism to the variability in clinical phenotypes of mast cell diseases.

In our *in vivo* model, expression of Kit D814V and simultaneous deletion of Tet2 in mature mast cells in the skin using the Mcpt5-Cre driver appeared to cause a more aggressive disease phenotype, but the difference between genotypes did not reach statistical significance. We conclude that the two lesions together have only modest transforming ability when expressed in the mature mast cell compartment compared with BM stem cells/progenitors. Therefore, data presented here validate loss of Tet2 as a molecular event specifically associated with more aggressive types of SM, which originate from the BM progenitor compartment and cause infiltration of various organs. Importantly, we also demonstrate here that the presence of Tet2 mutations alone is not sufficient to initiate mastocytosis, nether in the bone marrow compartment, nor in mature mast cells in the skin.

In this study, we found that all mice that succumbed to an ALL-like malignancy demonstrated an increase in cutaneous mast cells, suggesting that the Kit D814V transgenic model could serve as a model for ALL associated with SM. In humans, systemic mastocytosis with associated clonal hematological non-mast cell lineage diseases (SM-AHNMD) is a heterogeneous clinical entity [35], [36], [37], with a variable presence of *KIT D816V* in the malignant non-mast cell clone. Although ALL associated with SM has been reported only in sporadic cases [38], [39], the presence of the *KIT D816V* mutation has been documented in lymphocytes from patients with aggressive type of disease or MCL [5], and previous retroviral and transgenic models have demonstrated that *Kit D814*V has a preferential transforming potential on B cell precursors [40], [41].

Deletion of Tet2 did not influence initiation of Kit D814V-driven ALL-like disease in our experiments. This may be explained by the very short latency of the malignant disease in our model. On the other hand, *TET2* mutations have not been reported so far in human B-ALL at diagnosis [42]. However, in our experiments loss of both copies of Tet2 shortened survival of secondary recipients, suggesting that loss of Tet2 plays a role in progression of B-ALL initiated by Kit D814V. It will be interesting to analyze a larger cohort of adult patients with B-ALL (where samples at diagnosis and relapse are available), to examine the role of TET2 in this disease.

DASA has partial efficacy in patients with *KIT D816V* positive SM, and its tolerability profile frequently limits dose escalations in the clinical setting [9]. DAC and 5-azacytidine (5-AZA), which are effective for MDS and AML [43], have been shown as single agents to induce apoptosis in a human *KIT D816V* positive cell line (HMC-1.2) at high doses [44]. Recent evidence from both hematological and solid tumors has demonstrated that treatment of malignant cell lines with low doses of demethylating agents such as DAC and 5-AZA induces stable epigenetic remodeling of the treated cell genomes and induces apoptosis [45], [46]. These data suggest that low doses of these epigenetic drugs could be more effective than high doses. We found that low doses of DAC in combination with DASA are effective in inducing apoptosis and cell death in HMC-1.2 cells, and that the two-drug combination is more effective upon TET2 depletion. We also provide data suggesting that the combination of midostaurin (PKC412) and DAC works well *in vitro* on cell lines carrying the Kit activating mutation *D816V* and loss of TET2. As more clinical data become available on the efficacy and toxicity profile of midostaurin as a single agent in the treatment of ASM (10), our data provide an *in vitro* rationale to exploit the cooperation between this TKI and epigenetic modifiers. Additional studies are warranted to explore how TKIs and DAC act in combination and to investigate the effect of DAC on the epigenome of malignant mast cells. We believe that our findings may lead to new approaches to the treatment of patients with ASM harboring both *KIT D816V* and mutations in *TET2*.

## Supporting Information

Figure S1 **shRNA-mediated knock down of TET2 in HMC-1.2** **A**) Reduction in the level of TET2 mRNA was quantified using qRT-PCR and normalized to the housekeeping gene GAPDH. RNA was isolated from HMC-1.2 seven days after transduction. TET2 sh-1 and TET2 sh-3 were used for experiments described in the results section. Values are presented as fold change to ctr sh and represent means ±SEM (n = 3). The Ctr sh used for this and all subsequent experiments was the Luciferase G4 construct from Sigma-Aldrich (St. Louis, MO). **B**) Histogram plot demonstrating total 5-hmC content (quantified by intracellular flow staining) in HMC-1.2 cells upon KD of TET2. The grey filled curve represents the secondary Ab control, solid and dotted black lines indicate TET2 sh-1 and sh-3 and the dashed black line represents ctr sh. Shown is one of two independent experiments with similar results. **C**) Number of HMC-1.2 cells migrated in response to hSCF in an *in vitro* transwell migration assay. Bar graph represents average fold change in number of migrated HMC-1.2 transduced with TET2 sh-1 and sh-3 relative to ctr sh (n = 3, error bars represent SEM). No significant difference was observed among experimental groups.
(PDF)

Figure S2 **BM immunophenotype and competitive transplants in Mx1-Cre transgenic mice. A**) Total number of colonies formed in methylcellulose from Tet2^+/+^;Kit D814V, Tet2^+/−^;Kit D814V and Tet2^−/−^;Kit D814V animal at the initial density (1^st^ round) and after a second and third round of replating. **B**) Peripheral blood chimerism data on recipient animals transplanted with equal doses of whole bone marrow test cells (45.2) and supporting cells (45.1/45.2). Data show a significant repopulation advantage for both Tet2^+/+^;Kit D814V and Tet2^+/−^;Kit D814V at 16 and 20 weeks over competitor cells, with a more pronounced competitive advantage for Tet2^+/−^;Kit D814V 20 weeks after transplantation (*P<.05 Tet2^+/+^;Kit D814V vs. Tet2^+/−^;Kit D814V 45.2 donor derived cells at 20 weeks).
(PDF)

Figure S3 **Validation of pI:C-mediated deletion of the Kit D814V flox Stop cassette and the Tet2 targeted allele in Mx1-Cre transgenic animals.** **A**) Schematic view of the target allele in Kit D814V floxed animals. **B**) Schematic view of the target allele in Tet2 floxed animals. **C**) Kit D814V Stop deletion and Tet2 deletion PCR on genomic DNA extracted from BMMCs from induced animals. Position and size of wt, floxed and deleted alleles are shown. Numbers from 1 to 5 indicate the following genotypes: 1)Mx1-Cre, 2)Tet2^+/+^;Kit D814V, 3)Tet2^+/−^;Kit D814V, 4)Tet2^+/−^;Kit D814V, 5)Tet2^Fl/WT^;Kit D814V^Fl^. **D**) Percentage of BMMCs positive for Fcε but negative for c-Kit after 4 weeks in culture with IL-3. Single positive cells were 2.6±1.2 for the Tet2^+/+^;Kit D814V, 11.27±2.1 for the Tet2^+/−^;Kit D814V and 19.57±9.5 for the Tet^−/−^;Kit D814V group.*P<.05. **E**) qRT-PCR analysis of bone-marrow specific transcripts across genotypes. There was no significant difference in the level of carboxypeptidase 3 (Cpa-3), Il-4 and Tnfa mRNA/Gapdh across genotypes. Values are expressed as fold change to Tet2^+/+^;Kit D814V, and they all represent mean ±SEM (n = 3). Ns = not significant.
(PDF)

Figure S4 **Characterization of the ALL phenotype in diseased animals.** **A**) Representative H&E staining of peripheral blood smear, liver, spleen and bone marrow sections from a diseased animal. Scale bars represent 25 µm and 200 µm, respectively. **B**) Expression of B220 and CD19 on ALL blasts **C**) mRNA levels of Tet2 normalized to Gapdh mRNA in sorted blasts (data are expressed as fold changes relative to Tet2^+/+^;Kit D814V animals and represent means ± SEM (n = 3–4 animals/genotype)). **D**) Sequence analysis of cDNA from sorted blasts to verify the presence of the Kit D814V mutant allele in diseased animals. Data presented in A, B, D were based on one Tet2^+/−^;Kit D814V animal, but were reproduced in multiple animals across different genotypes.
(PDF)

Figure S5 **Knock-down of Tet2 enhances response of HMC-1.2 to midostaurin and decitabine.** HMC-1.2 cells were infected with two sh targeting TET2 (sh-1 and sh-3) and a control sh. Transduced cells were treated with decitabine or DMSO for 72 hours, then washed and treated with midostaurin (PKC412). Annexin V staining was performed 24 hours after PKC412 treatment was started. Bar graph indicates percentage of apoptotic (Annexin V^+^/7 AAD^−^) and dead cells (Annexin V^+^/7 AAD^+^) per treatment condition according to genotype. Pretreatment of cells with decitabine did not significantly increase the percentage of dead and apoptotic cells compared to treatment with PKC412 alone (*P* = .07, *P* = .1 and *P* = .3 for TET2 sh-1, TET2 sh-3 and ctr sh). However, the combination therapy worked significantly better in TET2 KD than in ctr sh cells (**P<.01 for TET2 sh-1 vs ctr sh, *P<.05 for TET2 sh-3 vs ctr sh). All values represent mean ±SEM (n = 3).
(PDF)

Table S1 **Sequence of the short hairpins used to target TET2.**
(PDF)

Table S2 **Incidence of ALL in primary Mx1-Cre transgenic mice.** A cohort of 6–12 mice per genotype was observed for 20 weeks after the last pI:C injection, and the number of mice with ALL was recorded. There was no significant difference in the incidence of ALL across genotypes.
(PDF)

Methods S1 **Supporting methods and references.**
(PDF)
