# Supplementary figures and images for: Loss of Function of TET2 Cooperates with Constitutively Active KIT in Murine and Human Models of Mastocytosis
Source: PLoS One. 2014 May 2;9(5):e96209. doi: 10.1371/journal.pone.0096209 (PMC4008566; doi:10.1371/journal.pone.0096209)

Figure S1

**A**

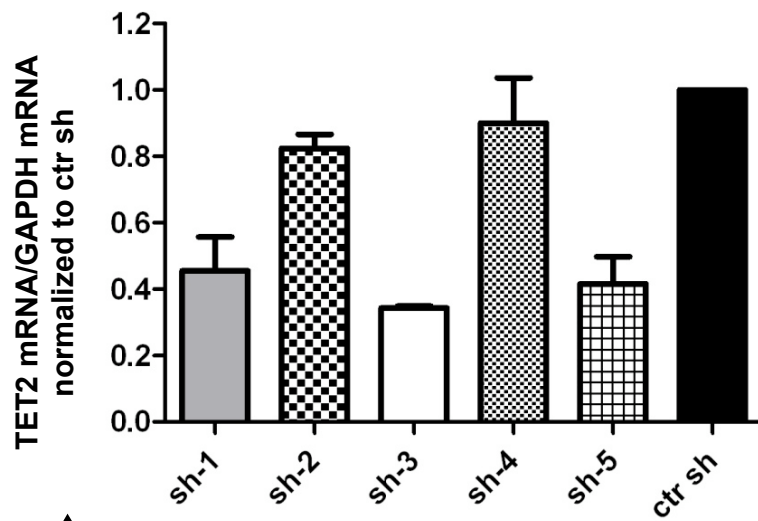

**B**

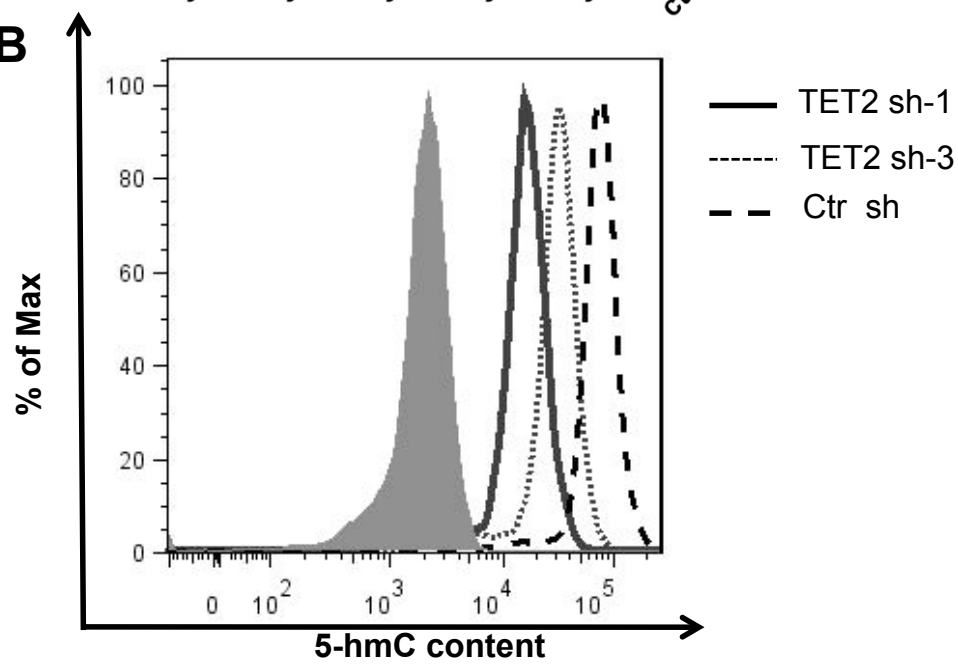

**C**

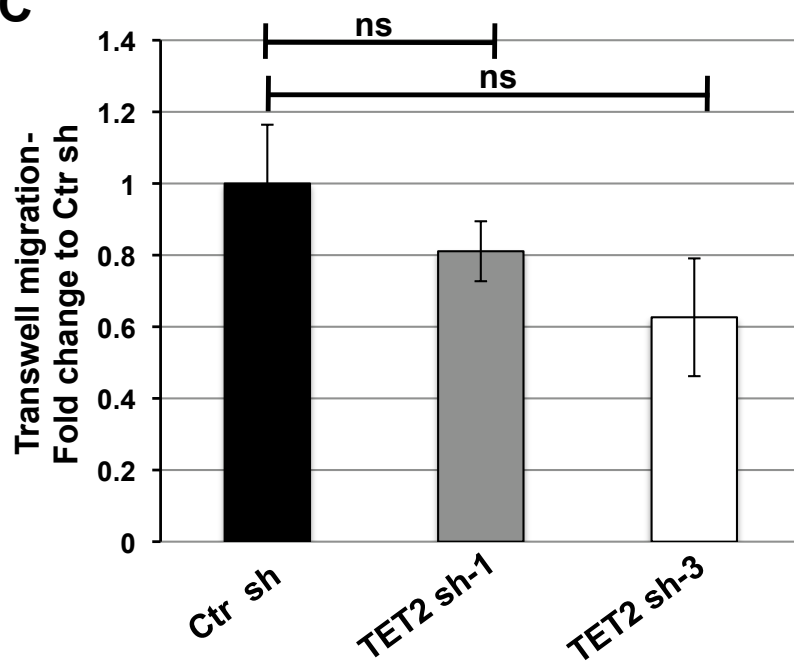

Supplement: Figure S1 — shRNA-mediated knock down of TET2 in HMC-1.2 A) Reduction in the level of TET2 mRNA was quantified using qRT-PCR and normalized to the housekeeping gene GAPDH. RNA was isolated from HMC-1.2 seven days after transduction. TET2 sh-1 and TET2 sh-3 were used for experiments described in the results section. Values are presented as fold change to ctr sh and represent means ±SEM (n = 3). The Ctr sh used for this and all subsequent experiments was the Luciferase G4 construct from Sigma-Aldrich (St. Louis, MO). B) Histogram plot demonstrating total 5-hmC content (quantified by intracellular flow staining) in HMC-1.2 cells upon KD of TET2. The grey filled curve represents the secondary Ab control, solid and dotted black lines indicate TET2 sh-1 and sh-3 and the dashed black line represents ctr sh. Shown is one of two independent experiments with similar results. C) Number of HMC-1.2 cells migrated in response to hSCF in an in vitro transwell migration assay. Bar graph represents average fold change in number of migrated HMC-1.2 transduced with TET2 sh-1 and sh-3 relative to ctr sh (n = 3, error bars represent SEM). No significant difference was observed among experimental groups. (PDF) [file pone.0096209.s001.pdf]

A

Figure S2

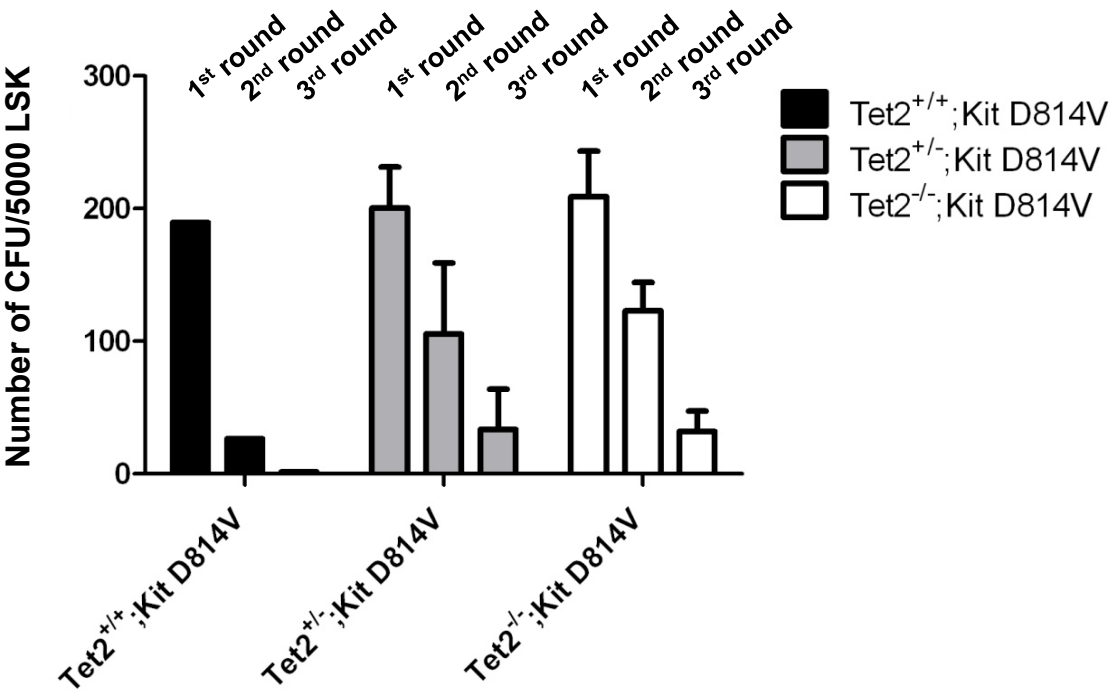

B

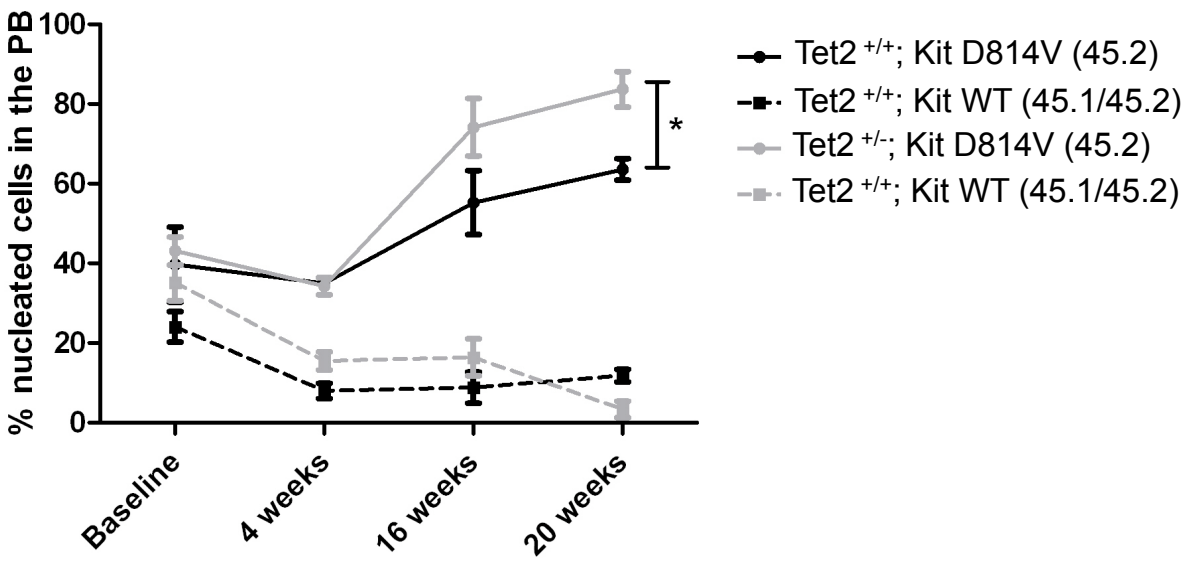

Supplement: Figure S2 — BM immunophenotype and competitive transplants in Mx1-Cre transgenic mice. A) Total number of colonies formed in methylcellulose from Tet2+/+;Kit D814V, Tet2+/−;Kit D814V and Tet2−/−;Kit D814V animal at the initial density (1st round) and after a second and third round of replating. B) Peripheral blood chimerism data on recipient animals transplanted with equal doses of whole bone marrow test cells (45.2) and supporting cells (45.1/45.2). Data show a significant repopulation advantage for both Tet2+/+;Kit D814V and Tet2+/−;Kit D814V at 16 and 20 weeks over competitor cells, with a more pronounced competitive advantage for Tet2+/−;Kit D814V 20 weeks after transplantation (*P<.05 Tet2+/+;Kit D814V vs. Tet2+/−;Kit D814V 45.2 donor derived cells at 20 weeks). (PDF) [file pone.0096209.s002.pdf]

**Figure S3**

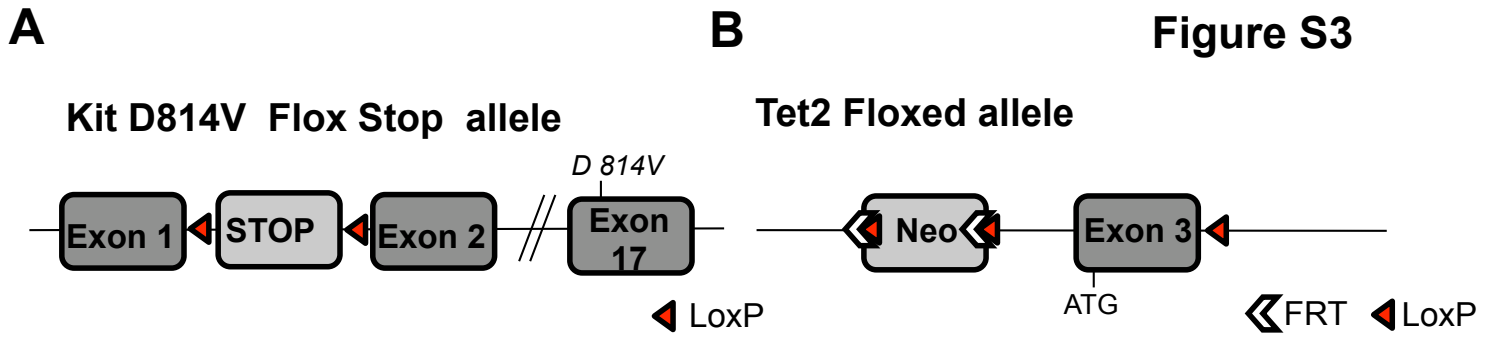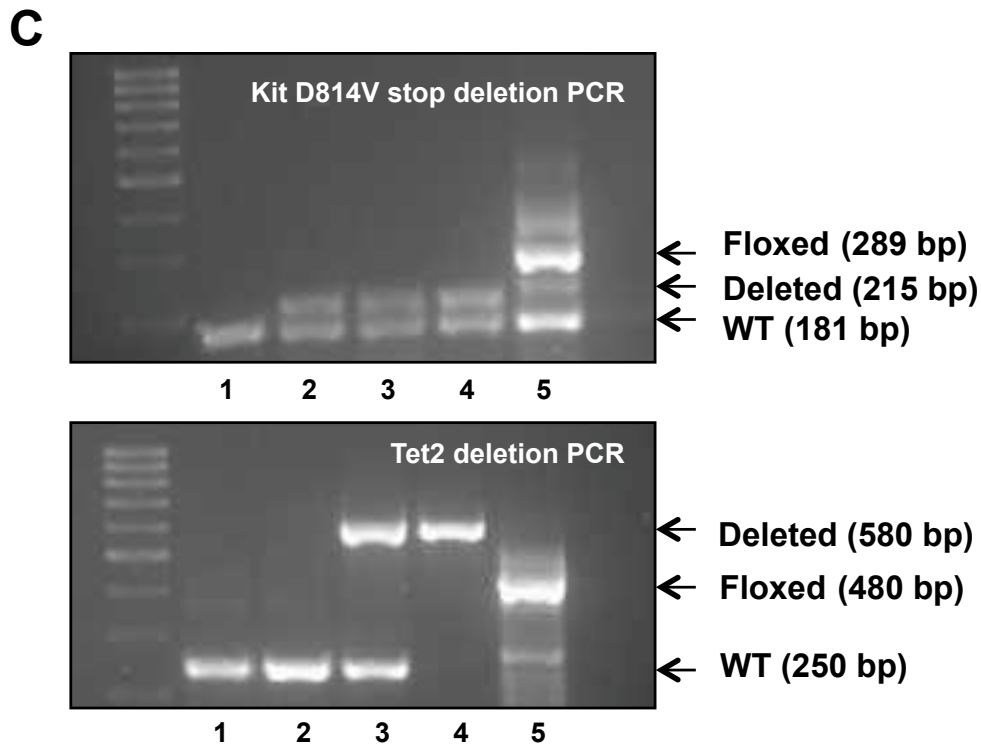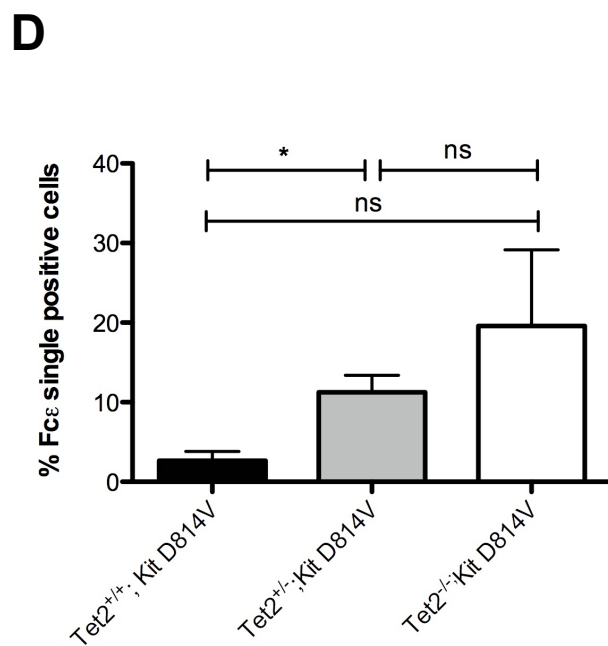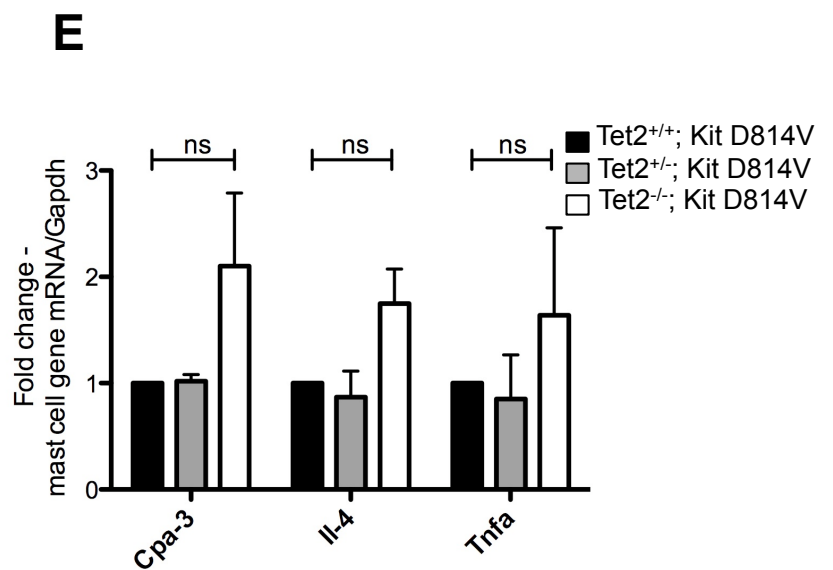

Supplement: Figure S3 — Validation of pI:C-mediated deletion of the Kit D814V flox Stop cassette and the Tet2 targeted allele in Mx1-Cre transgenic animals. A) Schematic view of the target allele in Kit D814V floxed animals. B) Schematic view of the target allele in Tet2 floxed animals. C) Kit D814V Stop deletion and Tet2 deletion PCR on genomic DNA extracted from BMMCs from induced animals. Position and size of wt, floxed and deleted alleles are shown. Numbers from 1 to 5 indicate the following genotypes: 1)Mx1-Cre, 2)Tet2+/+;Kit D814V, 3)Tet2+/−;Kit D814V, 4)Tet2+/−;Kit D814V, 5)Tet2Fl/WT;Kit D814VFl. D) Percentage of BMMCs positive for Fcε but negative for c-Kit after 4 weeks in culture with IL-3. Single positive cells were 2.6±1.2 for the Tet2+/+;Kit D814V, 11.27±2.1 for the Tet2+/−;Kit D814V and 19.57±9.5 for the Tet−/−;Kit D814V group.*P<.05. E) qRT-PCR analysis of bone-marrow specific transcripts across genotypes. There was no significant difference in the level of carboxypeptidase 3 (Cpa-3), Il-4 and Tnfa mRNA/Gapdh across genotypes. Values are expressed as fold change to Tet2+/+;Kit D814V, and they all represent mean ±SEM (n = 3). Ns = not significant. (PDF) [file pone.0096209.s003.pdf]

**Figure S4**

**A**

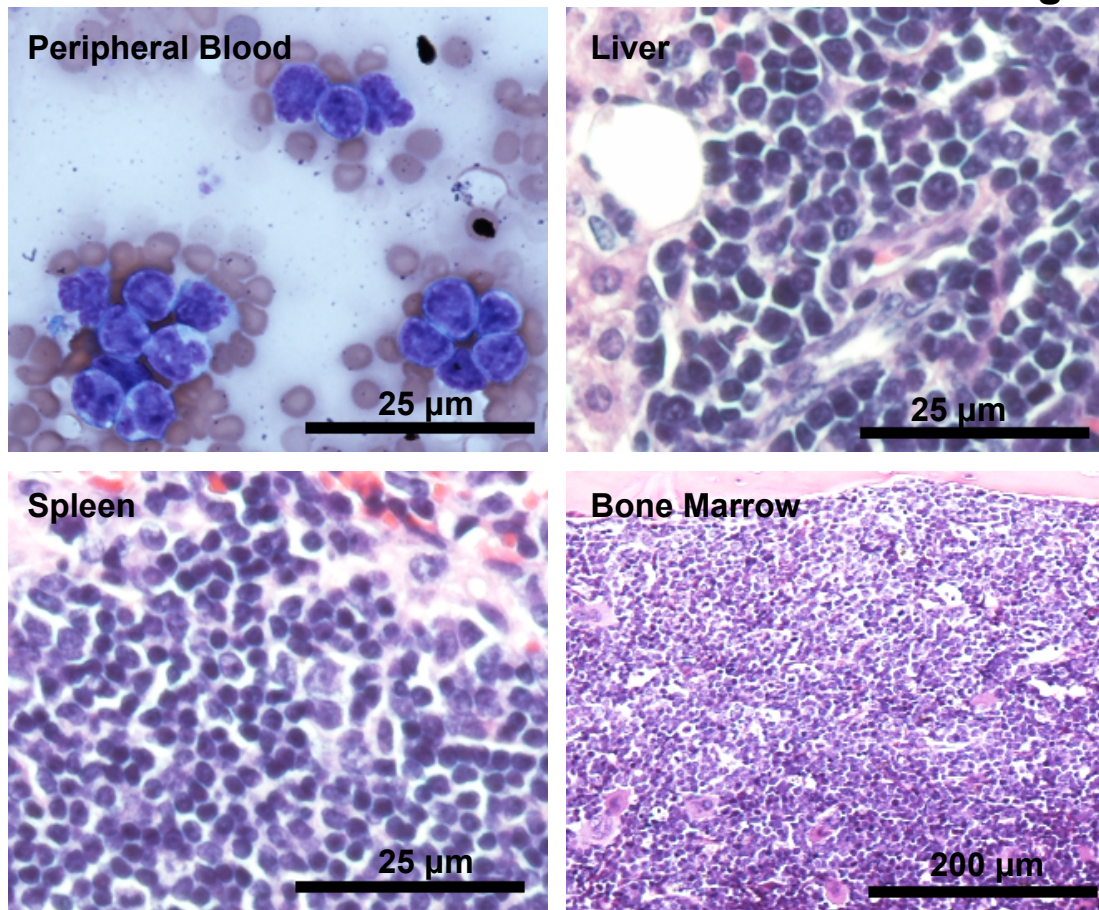

**B**

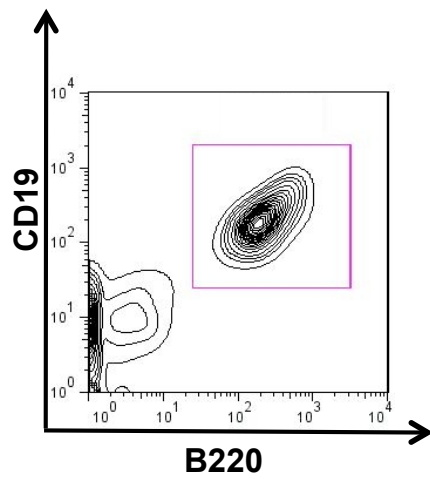

**C**

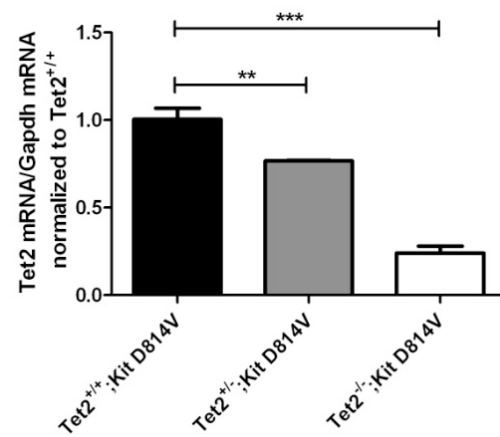

**D**

T C G G G C T A G C C A G A G A G T C A G G A A

WT: CCAGAGAC

MUT: CCAGAGTC

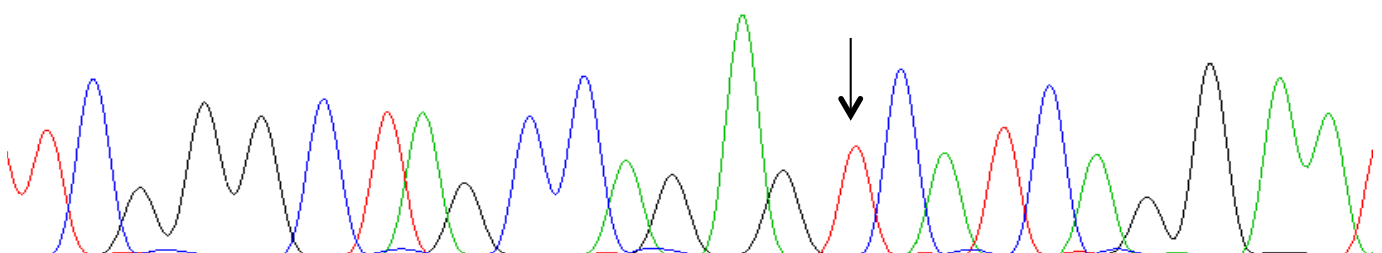

Supplement: Figure S4 — Characterization of the ALL phenotype in diseased animals. A) Representative H&E staining of peripheral blood smear, liver, spleen and bone marrow sections from a diseased animal. Scale bars represent 25 µm and 200 µm, respectively. B) Expression of B220 and CD19 on ALL blasts C) mRNA levels of Tet2 normalized to Gapdh mRNA in sorted blasts (data are expressed as fold changes relative to Tet2+/+;Kit D814V animals and represent means ± SEM (n = 3–4 animals/genotype)). D) Sequence analysis of cDNA from sorted blasts to verify the presence of the Kit D814V mutant allele in diseased animals. Data presented in A, B, D were based on one Tet2+/−;Kit D814V animal, but were reproduced in multiple animals across different genotypes. (PDF) [file pone.0096209.s004.pdf]

Figure S5

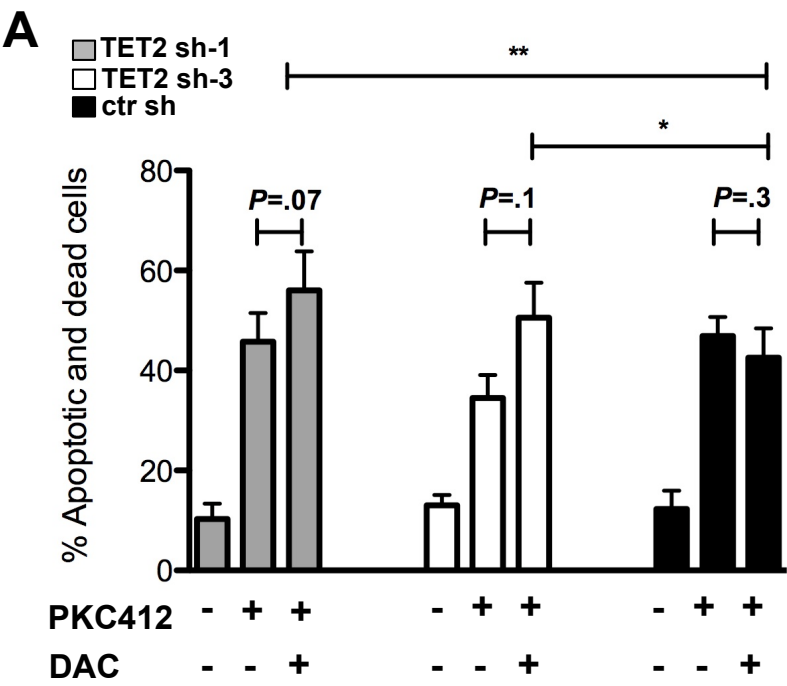

Supplement: Figure S5 — Knock-down of Tet2 enhances response of HMC-1.2 to midostaurin and decitabine. HMC-1.2 cells were infected with two sh targeting TET2 (sh-1 and sh-3) and a control sh. Transduced cells were treated with decitabine or DMSO for 72 hours, then washed and treated with midostaurin (PKC412). Annexin V staining was performed 24 hours after PKC412 treatment was started. Bar graph indicates percentage of apoptotic (Annexin V+/7 AAD−) and dead cells (Annexin V+/7 AAD+) per treatment condition according to genotype. Pretreatment of cells with decitabine did not significantly increase the percentage of dead and apoptotic cells compared to treatment with PKC412 alone (P = .07, P = .1 and P = .3 for TET2 sh-1, TET2 sh-3 and ctr sh). However, the combination therapy worked significantly better in TET2 KD than in ctr sh cells (**P<.01 for TET2 sh-1 vs ctr sh, *P<.05 for TET2 sh-3 vs ctr sh). All values represent mean ±SEM (n = 3). (PDF) [file pone.0096209.s005.pdf]
