# Supplementary material for: Loss of Function of TET2 Cooperates with Constitutively Active KIT in Murine and Human Models of Mastocytosis
Source: PLoS One. 2014 May 2;9(5):e96209. doi: 10.1371/journal.pone.0096209 (PMC4008566; doi:10.1371/journal.pone.0096209)
Supplement: Table S1 — Sequence of the short hairpins used to target TET2. (PDF) [file pone.0096209.s006.pdf]

Table S1

TET2 sh-1	CCGGGCCAAGTCATTATTTGACCATCTCGAGATGGTCA AATAATGACTTGGCTTTTTTG
TET2 sh-3	CCGGCCTCAGAGATATTGTGGGTTTCTCGAGAAACCCA CAATATCTCTGAGGTTTTTTG
