# Supplementary material for: Loss of Function of TET2 Cooperates with Constitutively Active KIT in Murine and Human Models of Mastocytosis
Source: PLoS One. 2014 May 2;9(5):e96209. doi: 10.1371/journal.pone.0096209 (PMC4008566; doi:10.1371/journal.pone.0096209)
Supplement: Table S2 — Incidence of ALL in primary Mx1-Cre transgenic mice. A cohort of 6–12 mice per genotype was observed for 20 weeks after the last pI:C injection, and the number of mice with ALL was recorded. There was no significant difference in the incidence of ALL across genotypes. (PDF) [file pone.0096209.s007.pdf]

Table S2

Genotype	Total number of mice	Mice with ALL	%
$Tet2^{+/+}$;Kit D814V	12	7	58.3%
$Tet2^{-/+}$;Kit D814V	17	10	58.8%
$Tet2^{-/-}$;Kit D814V	6	3	50%
