# Supplementary material for: Loss of Function of TET2 Cooperates with Constitutively Active KIT in Murine and Human Models of Mastocytosis
Source: PLoS One. 2014 May 2;9(5):e96209. doi: 10.1371/journal.pone.0096209 (PMC4008566; doi:10.1371/journal.pone.0096209)
Supplement: Methods S1 — Supporting methods and references. (PDF) [file pone.0096209.s008.pdf]

SUPPORTING INFORMATION

Loss of function of TET2 cooperates with constitutively active KIT in murine and human models of mastocytosis.

Serena De Vita, Rebekka K. Schneider, Michael Garcia, Jenna Wood, Mathilde Gavillet, Benjamin L. Ebert, Alexander Gerbaulet, Axel Roers, Ross L. Levine, Ann Mullally, David A. Williams

SUPPORTING METHODS AND REFERENCES

Transduction of HMC-1.2 with sh-RNA

Short hairpins (sh) targeting *TET2* and a control hairpin in a pLKO lentiviral backbone carrying GFP as a marker (TRC19) were obtained from the RNAi consortium library of the Broad Institute [1]. Sequences of the targeting sh are available in Table S1. Lentiviral particle preparation and cell transductions were performed according to our published protocols [2]. GFP-positive cells were isolated by cell sorting on a FACSAria (BD Biosciences, San Jose, CA).

Reagents

Stock solutions of dasatinib, PKC412 (both from LC laboratories, Woburn, MA), and decitabine (5-aza-2'-deoxycytidine, Sigma-Aldrich, St. Louis, MO) were prepared in DMSO and used at the working dilutions indicated in the results section. Antibodies used for western blotting included: p-Y719-c-Kit, c-Kit, p-Y694-STAT5, STAT5, SRC and cleaved CASPASE 3 from Cell Signaling (Danvers, MA), p-Y418-SRC from Invitrogen (Carlsbad, CA) and β-Actin from Sigma-Aldrich. Purity of BMMC cultures was assessed by surface

antigen staining using flow cytometry antibodies against FcεRI and c-Kit (both antibodies from eBioscience, San Diego, CA).

5-hmC staining

Cells were fixed and permeabilized with the BD fix/perm buffer. Following treatment with DNAse (also from BD), cells were stained with a primary unconjugated 5-hmC antibody (Active Motif, Carlsbad, CA) and a secondary Alexa Fluor 647 antibody from Cell Signaling (Danvers, MA) [3].

***In vitro* transwell migration assay**

For in vitro transwell assays, 8x 10^4 transduced HMC-1.2 cells were plated in the upper well of a transwell chamber with 8-μm pore filters (Corning, Corning, NY) in triplicates. Human recombinant SCF (Peprotech, Rocky Hill, NJ) was placed in the lower chambers at the concentration of 100 ng/mL. After three hours of incubation at 37°C in 5% CO2, migrated cells in the bottom chamber were enumerated using Countbright Beads (Invitrogen, Carlsbad, CA) according the manufacturer's instructions and analyzed by FACS (LSR II, BD Biosciences, San Jose, CA).

qRT-PCR and western blotting

Total RNA was extracted using the RNeasy Plus mini Kit from Qiagen (Valencia, CA). cDNA was synthesized using the Superscript II enzyme from Invitrogen following the manufacturer's instructions. qRT-PCR was performed on a 7500 Real Time PCR system (Applied Biosystems, Woburn, MA) using the relative quantification method. Primer sequence is available upon

request. Protein lysates were prepared using RIPA buffer supplemented with protease inhibitors (Roche, Indianapolis, IN) and phosphatase inhibitors (Thermo Scientific, Waltham, MA). Western blot analysis was performed using standard methods [4].

gDNA extraction and genotyping

gDNA was extracted from tails, cells suspensions and peripheral blood of animals using the DNeasy Blood & Tissue Kit from Qiagen (Valencia, CA) according to the manufacturer's instruction. PCR for Kit D814V genotyping and deletion was performed using the following primers: Kit Int1-2 Fwd: 5'-GAAAGAGCGGCAGACAAGAG; Int1-2 Typing Rev: 5'-TGAGGTCTCAGCTCAGGTG; Int1-2 Neo Stop typing Rev: 5'-AGAGGCCACTTGTGTAGCGC. Primers for Tet2 genotyping were: Tet2 Fwd 5'-GTGAAGGATGCAATCCAGGT, Tet2 Rev: CCAAGACCTCCCTGATGCTA. Primers for Tet2 deletion were: Tet2F2: 5'-AAGAATTGCTACAGGCCTGC; Tet2R2: 5'-TTCTTTAGCCCTTGCTGAGC; Tet2 loxP3: 5'-TAGAGGGAGGGGGC. Before each experiment reported in this manuscript, we verified derepression of the Kit D814V transgenic allele by Cre-mediated deletion of the Flox-Stop allele, and deletion of the floxed Tet2 allele(s) by PCR on genomic DNA isolated from peripheral blood collected one week after the last pI:C injection.

Mx1-Cre genotyping primers were: Cre-Fwd: 5'-CGTATAGCCGAAATTGCC; CRE2-1: 5'-CAAAACAGGTAGTTATTC. Mcpt5-Cre genotyping primers were: Mcpt5-CreUP: 5'-ACAGTGGTATTCCCGGGGAGTGT; CreSeq1b-DO:5'-

GTCAGTGCGTTCAAAGGCCA; Mcpt5-Ex1-DO3: 5'-GCTTTGGTGCTGGAACCCAGGA.

CFU assay

LSK from 20 weeks animals were isolated as previously described [5]. Sorted cells were seeded at the density of 5000 cells/replicate into methylcellulose medium (MethoCult supplemented with cytokines from STEMCELL Technologies, Vancouver, Canada). After 10 days, colonies were scored, then harvested, counted and replated for sequential rounds.

Competitive transplants

Bone marrow cells were harvested from Tet2 $^{WT/WT}$;Kit D814V^{Fl};Mx1-Cre and Tet2 $^{Fl/WT}$; Kit D814V^{Fl};Mx1-Cre mice (CD45.2$^+$), counted and mixed with an equal number of supporting cells from CD45.1$^+$/CD45.2$^+$ donor mice. Cells were injected into lethally irradiated (1100 cGy split in two doses) recipients (CD45.1$^+$) by tail vein injection. Chimerism was evaluated by flow cytometry on total nucleated cells in the peripheral blood at 4 weeks after transplant (baseline, pre-pI:C). Mice were then treated with three doses of pI:C, and chimerism followed over time up to 20 weeks after transplantation.

Leukemic cells immunophenotype and sorting strategy

ALL blasts were identified by co-expression of B220 and CD19 (all antibodies used for flow staining were from eBioscience (San Diego, CA)). Cells positive for the two markers were sorted on a FACSAria (BD Biosciences, San Jose, CA) and frozen down. Cells were then defrosted, counted and transplanted at

different doses into sublethally irradiated (350 cGy) congenic recipients. Recipient mice (C57BL/6J) were 6-8 weeks old and were purchased from The Jackson Laboratory (Bar Harbor, ME).

Kit D814V cDNA sequencing

cDNA synthesized from RNA isolated from bone marrow of animals with ALL was sequenced with the following primers: Kit Fwd: 5'-CCTCGCCTCCAAGAATTGT; Kit Rev: 5'-AGGAGAAGAGCTCCCAGA.

SUPPLEMENTARY REFERENCES

1. Moffat J, Grueneberg DA, Yang X, Kim SY, Kloepfer AM, et al. (2006) A lentiviral RNAi library for human and mouse genes applied to an arrayed viral high-content screen. Cell 124: 1283-1298.

2. Muller LU, Milsom MD, Kim MO, Schambach A, Schuesler T, et al. (2008) Rapid lentiviral transduction preserves the engraftment potential of Fanca(-/-) hematopoietic stem cells. Mol Ther 16: 1154-1160.

3. Chen C, Liu Y, Lu C, Cross JR, Morris JPt, et al. (2013) Cancer-associated IDH2 mutants drive an acute myeloid leukemia that is susceptible to Brd4 inhibition. Genes Dev 27: 1974-1985.

4. Milsom MD, Schiedlmeier B, Bailey J, Kim MO, Li D, et al. (2009) Ectopic HOXB4 overcomes the inhibitory effect of tumor necrosis factor-{alpha} on Fanconi anemia hematopoietic stem and progenitor cells. Blood 113: 5111-5120.

5. Dorrance AM, De Vita S, Radu M, Reddy PN, McGuinness MK, et al. (2013) The Rac GTPase effector p21-activated kinase is essential for hematopoietic stem/progenitor cell migration and engraftment. Blood 121: 2474-2482.
